# A Simple Fiber Bragg Grating-Based Sensor Network Architecture with Self-Protecting and Monitoring Functions

**DOI:** 10.3390/s110201375

**Published:** 2011-01-26

**Authors:** Chien-Hung Yeh, Chi-Wai Chow, Ping-Chun Wu, Fan-Gang Tseng

**Affiliations:** 1 Information and Communications Research Laboratories, Industrial Technology Research Institute (ITRI), Hsinchu 31040, Taiwan; 2 Department of Photonics and Institute of Electro-Optical Engineering, National Chiao Tung University, Hsinchu 30010, Taiwan; E-Mails: cwchow@faculty.nctu.edu.tw (C.-W.C.); depew007@yahoo.com.tw (P.-C.W.); 3 Department of Engineering and System Science, National Tsing Hua University, Hsinchu 30013, Taiwan; E-Mail: fangang@ess.nthu.edu.tw

**Keywords:** FBG sensor, fault protection, passive ring architecture

## Abstract

A novel fiber Bragg grating (FBG)-based passive sensor architecture, which can be used to protect the fiber cut and monitor the multiple sensors simultaneously, is proposed and experimentally demonstrated. Here, we employ a wavelength-tunable erbium-doped fiber (EDF) laser scheme with 25 km cavity length acting as the detecting light source in central office (CO). Each FBG sensor, serving as a feedback element, is used in proposed sensor architecture. By tuning the tunable bandpass filter (TBF) placing inside cavity to match the corresponding Bragg wavelength of FBG over the amplification bandwidth, we can retrieve the related wavelength lasing for the FBG sensing and monitoring simultaneously. Moreover, the survivability and capacity of the passive FBG sensor architecture can be also enhanced.

## 1. Introduction

Using fiber Bragg gratings (FBG) to serve as the fiber-optic sensors are important studies in the field of optical sensing applications [1–3]. When strain or temperature variations are imposed on the FBG sensor, the Bragg wavelength will drift due to the index change of the FBG, and thus the detected wavelength will also shift. For distributed fiber sensors in intelligent sensing systems, the FBG-based sensor has been studied and identified as an important sensing element for sensing the change of temperature and strain [1–5]. Hence, a large-scale FBG sensor system could be built and achieved easily by a multiplexing method [6–8].

During the strain sensing, if the payload applied on the FBG is over its limitation, FBG breakage will occur. Furthermore, the fiber-fault of FBG-based sensing network also could affect the survivability and reliability. As a result, how to improve and enhance the reliability and survivability of sensor network becomes the essential issue for further study. To ensure the survivability of an FBG sensor system against fiber faults, a few self-protection schemes have been reported [9,10]. However, these methods required optical switches (OSWs) in each remote node (RN) to re-route the sensing path for detecting and protecting the fiber fault. It was hard to control and judge the direction of OSWs in each RN and also increased the complexity and cost of FBG-sensor networks. In addition, even though these FBG sensor systems were passive sensing networks in multi-ring schemes in the past studies [1,2], these ring sensor architectures required many numbers of RNs, consisting of optical coupler (OCP), to produce multi-ring configurations and result in the huge fiber connect points to reduce the power budget after run trip transmission.

In this study, a self-protected passive FBG sensor network, which enhances the reliability and survivability in long-reach fiber distance, is proposed and experimentally investigated. The sensing mechanism is based on a 25 km cavity length erbium-doped fiber (EDF) ring laser for detecting the multiple FBG sensors in the network. And we only use an optical coupler (OCP) on RN to produce the multi-ring configuration. The advantage of the proposed fiber laser scheme for detecting and monitoring the FBG sensors in the long distance self-protected scheme can facilitate highly reliable and survivable operation. Besides, the long distance sensing system can be integrated in fiber access network to reduce the cost of sensor infrastructure in the future.

## 2. Experiment and Discussion

**Figure 1.**
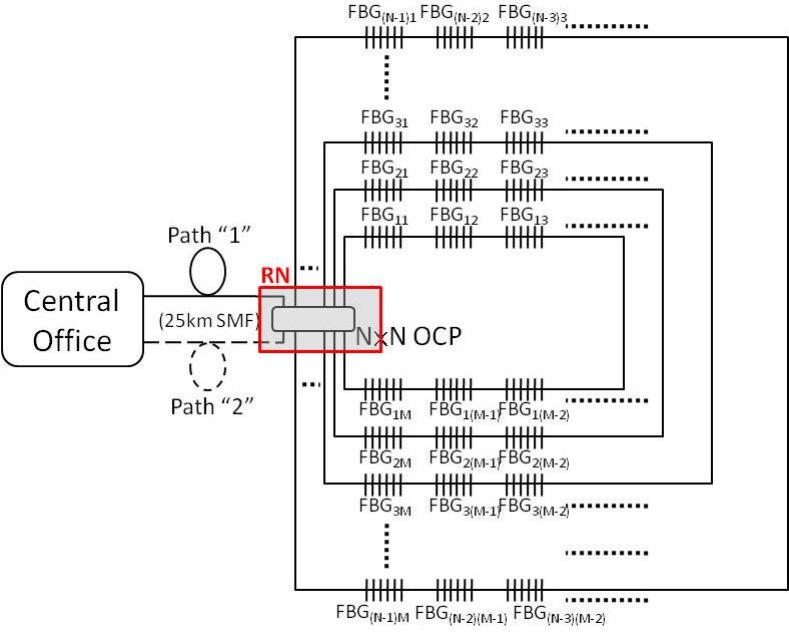
Proposed long distance FBG sensor system in passive multi-ring architecture. SMF: single-mode fiber; OCP: optical coupler; FBG: fiber Bragg grating; RN: remote node.

The proposed LR self-healing FBG-based sensor system consisted of a central office (CO) and multiple sensing networks, as shown in Figure 1. Here, two 25 km long single-mode fibers (SMFs) were used to connect to the CO and a *N* × *N* optical coupler (OCP), which was located at remote node (RN). The upper (path “1”) and lower SMFs (path “2”) were used to serve as the feeder fibers for sensing signal transmission. The *N* × *N* OCP could produce *N* – 1 fiber ring architectures and each ring scheme could use *M* FBGs for system sensing. A wavelength-tunable laser (WTL) source was used to detect and monitor the FBG sensors in the CO. For the initial deployment of the sensing network, the power budget (*i.e.*, the power of the laser source and the split-ratio of the sensing network) should be carefully considered. If the power budget is not enough due to high split ratio, fiber amplifiers between CO and RN can be used to solve this issue. Besides, according to current passive access network standards [11], this proposed 25 km long sensor system can be also integrated in the fiber access system to enhance the use of capacity fiber and reduce the cost of sensor infrastructure.

**Figure 2.**
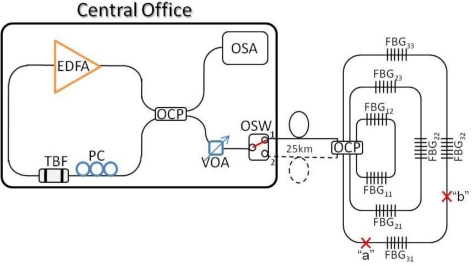
Experimental setup of proposed long distance self-healing FBG-based sensor system.

To realize and perform the proposed FBG-based sensor network, the experimental setup was based on a simplified version as illustrated in Figure 2. The CO was constructed by a WTL, a 1 × 2 OSW and an optical spectrum analyzer (OSA). In this measurement, the WTL was consisted of an erbium-doped fiber amplifier (EDFA), a tunable bandpass filter (TBF), a 4 × 4 OCP, a polarization controller (PC), a variable optical attenuator (VOA). The 980 nm pumping laser of EDFA operated at 215 mA and the saturated output power of the EDFA was around 16 dBm at 1,530 nm. The tuning range, insertion loss and 3 dB bandwidth of TBF used was 36 nm (1,526 to 1,562 nm), <0.7 dB and 0.3 nm, respectively. The PC and VOA were used to control and adjust the polarization status and maintain the maximum output power. Thereby, the 4 × 4 OCP would introduce three ring sensing networks. And the two feeder fibers were 25 km long. In the experiment, the sensor network has eight FBGs, which could be used for the strain and temperature sensing applications. Moreover, the Bragg wavelengths of these FBGs, which have different reflectivity, were 1,527.6 (λ_11_), 1,528.9 (λ_12_), 1,532.9 (λ_21_), 1,536.7 (λ_22_), 1,538.4 (λ_23_), 1,541.7 (λ_31_), 1,546.0 (λ_32_) and 1,556.0 nm (λ_33_), respectively.

The proposed WTL scheme in CO contained the C-band erbium-doped gain operation. And the passband of the TBF, using the inside gain cavity, was scanned to match the corresponding Bragg wavelength of FBG. Each FBG element served as the reflected sensor head and was connected as the part of cavity via a 25 km long fiber. Due to the inclusion of the TBF within the cavity loop, the lasing wavelength would be generated only when the filter passband was aligned in accordance with one of the FBG elements. In normal operation, when the TBF was set at one of the Bragg wavelength of the FBG, a long cavity fiber ring laser will be formed, and a lasing wavelength will be detected at the OSA located in the CO, with a 0.05 nm resolution.

**Figure 3.**
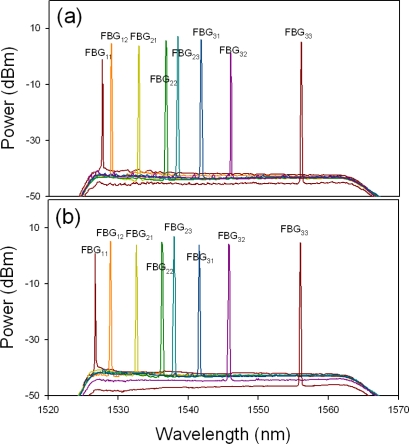
Output wavelengths of the proposed FBG-based sensor system from 1,527.6 to 1,556.0 nm via the **(a)** working and **(b)** protecting fiber links.

Initially, the OSW of CO was located on position “1” to connect to the sensor network via the upper feeder fiber (path “1”) for detecting and monitoring the eight FBG sensors used, as illustrated in Figure 2. Here, Figure 3(a) shows the reflected wavelengths of the proposed FBG-based sensor network from 1,527.6 to 1,556.0 nm (λ_11_ to λ_33_) via upper fiber. Here, we can observe eight lasing wavelengths in Figure 3(a) using the proposed WTL scheme during the TBF scanning. Of course, we could also connect the FBG sensor network for via the lower feeder fiber (path “2”), when the 1 × 2 OSW was switched to position “2”, as shown in Figure 2. Thus, Figure 3(b) presents the retrieved output wavelengths of the eight FBG sensors system via the lower feeder fiber. As shown in Figure 3(a) and Figure 3(b), no matter from which fibers to transmit the detecting signal to monitor FBG sensors, the measured reflective wavelengths are the same. However, the obtained wavelength λ_11_ of Figure 3(b) is slightly drifted due to the different forward and backward reflected spectrum of FBG_11_. As a result, we can switch the direction of OSW for connecting the upper or lower feeder fibers to monitor the status of FBG sensors and each ring fiber simultaneously.

**Figure 4.**
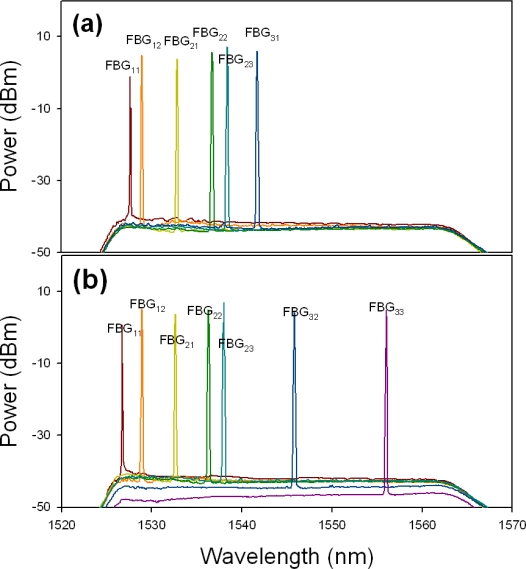
Output wavelengths of the proposed FBG-based sensor system *via* the **(a)** working and **(b)** protecting fiber links, when a cut occurs at “b” point in Figure 2.

**Figure 5.**
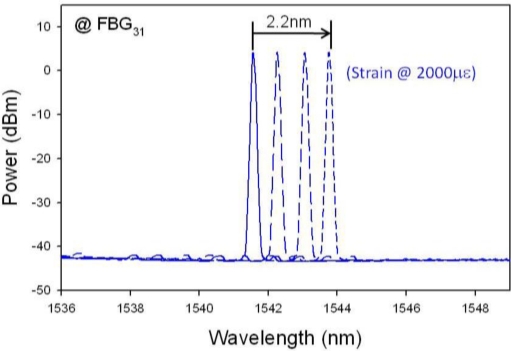
Reflected spectrum of FBG_31_ when the strain is applied.

In the first scenario, the fiber fault may happen inside the fiber ring network. Hence, if a fiber cut occurs at point “b”, as seen in Figure 2, the lasing wavelengths of λ_32_ and λ_33_ can be not measured *via* the upper feeder path, as shown in Figure 4(a). At this moment, the OSW will switch to position “2” to connect the lower feeder fiber for rescanning the FBG sensors. Thus, only the wavelength λ_31_ cannot be retrieved by the proposed WTL scheme, as illustrated in Figure 4(b). According to the measured results of Figure 4(a) and Figure 4(b), then we can locate and ensure the cut position among FBG_31_ and FBG_32_. Besides, if two fiber cuts occur at point “a” and “b” simultaneously in the sensor network, the lasing wavelengths of λ_31_, λ_32_ and λ_33_ via the paths “1” and λ_31_ via the path “2” cannot be measured by OSA, respectively. Hence, we can ensure the faults are among points “a” and “b”. Furthermore, when the external strain is applied on FBG_31_ before breaking, wavelength shifts can be observed, as illustrated in Figure 5. Here, the maximum strain shift of FBG_31_ is nearly 2.2 nm. When the temperature of FBG increases gradually, the reflected wavelength of the FBG will shift. In the proposed FBG sensing network, certain sub-ring systems could be used for the temperature sensing. Besides, some FBG sensors are used for both temperature-sensing as well as fiber-cut location detection. In the measurement, the maximum wavelength shift of FBG_31_ is ∼2.2 nm when the temperature of FBG_31_ change is around 68 °C. While the strain exceeds the limitation of FBG_31_, the FBG sensor will be broken. In such situation, if a fault occurs on FBG_31_, the measured lasing wavelengths in CO are the same comparing with the results of Figure 4. However, we can first observe the wavelength drift before FBG breakage. As a result, we can clearly know the differences of fiber-fault and FBG-fault in the proposed passive FBG sensor network.

**Figure 6.**
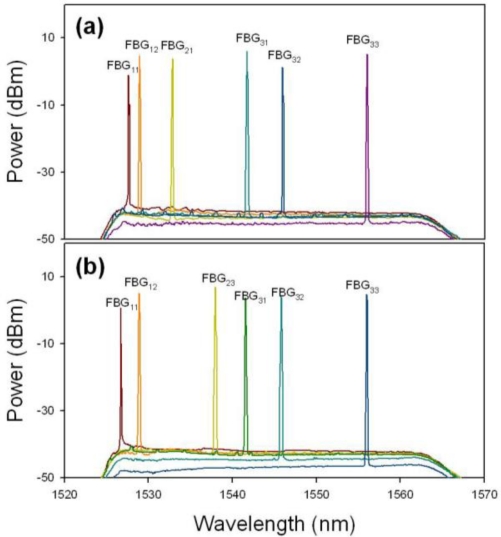
Measured output spectra of the sensing system via the **(a)** upper and **(b)** lower feeder fibers while a fault is on FBG_22_.

In addition, it is necessary to ensure that each FBG sensor was in good condition in a fiber sensor system. Thus, when the strain or temperature change was applied on each FBG sensor due to the environmental or artificial effects, the sensor will cause the Bragg wavelength shift. For the second scenario, while the payload of the FBG exceeds the limitation, the FBG would be broken. Here, the proposed ring sensor architecture also could find the position of the broken FBG. For example, if the FBG_22_ was broken in the proposed sensor network via the fiber path “1”, the lasing wavelengths of λ_22_ and λ_23_ would not be detected, as shown in Figure 6(a). Hence, to detect the disappeared FBG sensors, the CO would control the OSW to reconnect to the lower feeder fiber (path “2”) for sensing FBG. Thus, the measured output spectrum lacks the λ_21_ and λ_22_, as shown in Figure 6(b). Comparing the two output spectra of Figures 6(a) and 6(b), we can easily locate the fault is on FBG_22_. Moreover, for example, when the measured lasing spectra of the λ_11_, λ_12_, λ_21_, λ_22_ and λ_23_ via the fiber path “1”, and λ_11_ and λ_21_ via the fiber path “2” respectively, are not detected by the proposed sensor system, we can assure that two sensors of FBG_11_ and FBG_21_ are broken this time.

We can follow the above measurement methods to ensure when the sensing path is via the feeder fiber paths “1” and “2”. Initially, we can observe entire reflected wavelengths of FBG sensors via feeder path “1” when this sensing network is without FBG fault or fiber fault. When a fault is produced on FBGxy, first we can observe that the reflective-wavelength of measured FBGxy should be shift and then broken. Based on the measured result, we can realize the fault which is on FBG not on fiber. Besides, when the occurrence of fiber fault is produced, all the observed reflective-wavelengths are not change at this time. As a result, the proposed FBG sensor system not only can find out the fiber fault, but also detect and monitor the FBG sensors.

## 3. Conclusions

In summary, we have proposed and experimentally investigated a simple self-restored FBG based sensor ring system for long distance sensing. Besides, there is no active component in the FBG sensor architecture for cost reduction. The sensing mechanism is based on a 25 km cavity length erbium-doped fiber (EDF) ring laser for detecting the multiple FBG sensors in the network. In this experiment, three scenarios of fault detections were experimentally studied, showing that the sensing network survivability, reliability and capacity for the multiple sensors can be enhanced. In the future, this proposed sensing network can be integrated into a fiber access network for advanced applications.
